# Histamine Causes Pyroptosis of Liver by Regulating Gut-Liver Axis in Mice

**DOI:** 10.3390/ijms23073710

**Published:** 2022-03-28

**Authors:** Qiaoqiao Luo, Ruoyu Shi, Yutong Liu, Libo Huang, Wei Chen, Chengtao Wang

**Affiliations:** 1 Beijing Advanced Innovation Center for Food Nutrition and Human Health, Beijing Engineering and Technology Research Center of Food Additives, School of Food and Health, Beijing Technology and Business University, Beijing 100048, China; luoqiaoqiao7469@163.com (Q.L.); shiruoy@126.com (R.S.); liuyt128@126.com (Y.L.); 2 College of Animal Science and Technology, Shandong Agricultural University, Taian 271000, China; huanglibo@sdau.edu.cn

**Keywords:** gut microbiota, food ingredients, metabolic disease, health, metabolism, molecular mechanism

## Abstract

Huangjiu usually caused rapid-drunkenness and components such as β-benzyl ethanol (β-be), isopentanol (Iso), histamine (His), and phenethylamine (PEA) have been reported linked with intoxication. However, the destructive effect of these components on gut microbiota and liver is unclear. In this study, we found oral treatment of these components, especially His, stimulated the level of oxidative stress and inflammatory cytokines in liver and serum of mice. The gut microbiota community was changed and the level of lipopolysaccharide (LPS) increased significantly. Additionally, cellular pyroptosis pathway has been assessed and correlation analysis revealed a possible relationship between gut microbiota and liver pyroptosis. We speculated oral His treatment caused the reprogramming of gut microbiota metabolism, and increased LPS modulated the gut-liver interaction, resulting in liver pyroptosis, which might cause health risks. This study provided a theoretical basis for the effect of Huangjiu, facilitating the development of therapeutic and preventive strategies for related inflammatory disorders.

## 1. Introduction

The cultivation and use of fermented wines can be traced back to 7000 years ago. The typical production technology is via a solid or semi-solid brewing mode of ‘bilatera fermentation’ with single or multiple grains such as glutinous rice, sorghum, wheat, corn, and rice as raw materials and wheat koji as a saccharifying starter [1,2]. During the above open fermenting process, various strains of yeast, bacteria, fungi, mold and other strains inoculated naturally or cooperatively undergo glucose metabolism, protein metabolism, and lipid metabolism. At the same time, complex flavor compounds were produced [2]. A variety of fermented wines have been developed and consumed in Asian countries. Recently, fermented foods are of public interest and consumed more frequently in western countries because fermented foods, particularly fermented wine, have been proven to have health-promoting and protective effects.

Huangjiu is a nutritive brewing wine made from rice, millet, corn, and other grains through cooking, saccharification, fermentation, filtration, and frying [3], thus Huangjiu is a complex mixture composed of many chemical components besides ethanol [4]. Carbohydrates, amino acids, organic acids, lipids are the main functional components in Huangjiu [3]. However, there are compounds in Huangjiu that may adversely affect its flavor, taste, and even safety of it [5]. Rapid-drunkenness has always been one of the most important factors affecting the consumers’ choice of Huangjiu, which has seriously limited the development of the Huangjiu industry. It has been reported that compounds such as higher alcohols, biogenic amines, aldehydes, and phenols have been linked with intoxication and hangover, as they can aggravate the effect of ethanol in distilled liquors and red wine [6,7]. Sun et al. [6] used the *cryprinus carpio* model to analyze the intoxication effect of alcoholic beverages and to assess the impacts of Huangjiu components on intoxication and found that β-benzyl ethanol(β-be) and isopentanol (Iso) had the greatest positive effect on Huangjiu intoxication, followed by histamine (His) and phenethylamine (PEA). These substances resulted in a physical imbalance of *C. carpio* to varying degrees and undoubtedly were harmful to the health. However, the destructive effect of these substances on the gut microbiota as well as the liver has not been explored yet.

The gut-liver axis refers to the complex two-way interaction between the gastrointestinal tract and the liver through the biliary tract, portal vein, and systemic circulation [8]. The communication path is bidirectional; which means, substances produced by the liver enter into the intestine through the biliary tract and systemic circulation; meanwhile, the liver receives about two-thirds of its blood through the intestine via the portal vein [9]. Recently, more and more studies have shown that the gut-liver axis plays a crucial role in hepatic disorders [8]. The underlying molecular mechanism may be that the constitution of the gut microbiome and its metabolites affect the pathophysiology of the liver, such as short-chain fatty acids (SCFAs), bile acids (BAs), choline, and lipopolysaccharides (LPS) [10,11,12]. Meanwhile, the liver components can affect liver health via influencing the community and function of the gut microbiota [13]. Thus, it can be seen that changes in gut microbial composition and its metabolites can lead to changes in liver pathophysiology.

Pyroptosis is the pro-inflammatory programmed death of cells, and its intensity depends on the activity of cysteine aspartate-specific proteases (Caspases). Caspases construct self-inhibiting C-terminal fragment (CT) and functional N-terminal fragment (NT) by cutting gasdermin family proteins, which are transferred to the membrane and perforated, resulting in water infiltration, cell swelling, which releases inflammatory factors, and then turns into pyroptosis [14]. It is reported that pyroptosis is involved in the occurrence and development of various organ and tissue lesions, such as heart disease, atherosclerosis, gastrointestinal diseases, diabetes, especially liver cancer in recent years [15,16].

In this study, we intend to study the possible adverse effects of four main components in Huangjiu, especially histamine, on health, so as to have a fuller understanding of this Chinese traditional food and contribute to the development of Huangjiu industry. We gavaged mice with four main components in Huangjiu (β-be, Iso, His, and PEA), respectively, and studied the destructive effect on the liver. The level of oxidative stress and inflammatory cytokines in serum and liver increased significantly with the treatment. The composition and metabolism of gut microbiota have been changed. In addition, the cellular pyroptosis pathway in the liver has been assessed. It can be speculated from the results that the four components, especially His, caused liver damage via modulating the gut-liver axis in mice. Oral histamine treatment caused significant compositional changes of the gut microbiota, resulting in an increase of LPS, which subsequently triggered oxidative stress and inflammatory damage to the liver, leading to the final pyroptosis of the liver and may cause health risks.

## 2. Results

### 2.1. Effects of His, PEA, β-Be, and Iso on Body Weight, Liver Weight, and Level of Oxidative Stress in Serum and Liver of Mice

**Figure 1:**
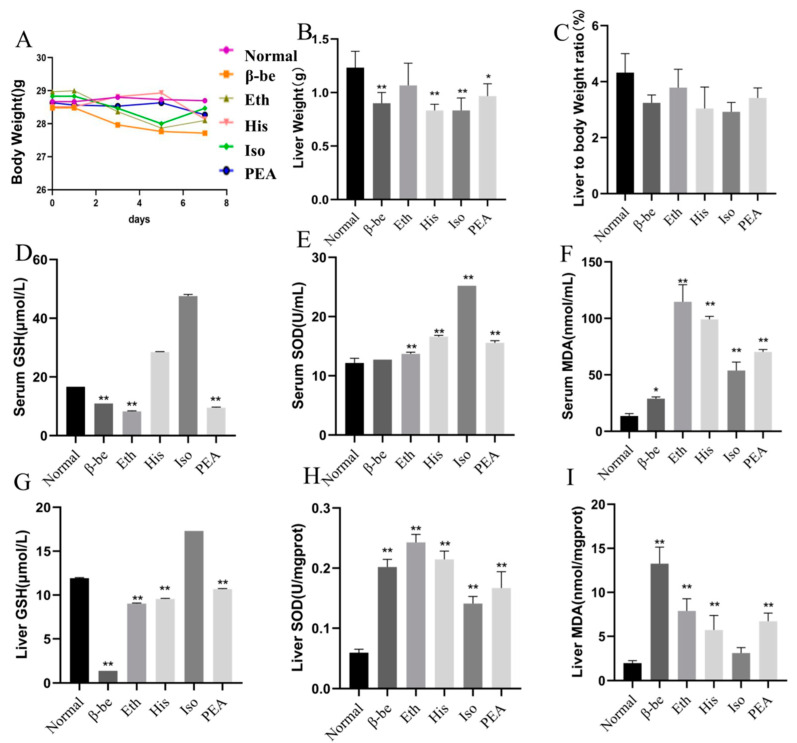
The effects of His, PEA, β-be, and Iso on body weight, liver weight, serum, and level of oxidative stress of mice. * *p* < 0.05, ** *p* < 0.01. (**A**) Changes in body weight. (**B**) Liver weight. (**C**) Liver to body weight ratio. (**D**) Serum GSH. (**E**) Serum SOD. (**F**) Serum MDA. (**G**) Liver GSH. (**H**) Liver SOD. (**I**) Liver MDA.

The results for the body weight, liver weight, serum, and liver biomarkers, such as MDA, GSH, SOD were presented in Figure 1A–I. There was no significance in body weight among groups (Figure 1A). Although the liver weight of β-be, His, Iso, and PEA groups was significantly decreased when compared with Normal (*p* < 0.01, Figure 1B), there was no significance between the ratios of liver weight to body weight (Figure 1C). The serum GSH, SOD, MDA of the Iso group were significantly higher than other groups (*p* < 0.01, Figure 1D–F). The liver GSH, SOD, MDA of His group were significantly lower than any other group (*p* < 0.01, Figure 1G–I).

### 2.2. Effects of His, PEA, β-Be, and Iso on Inflammatory Cytokines in the Liver and Serum

**Figure 2:**
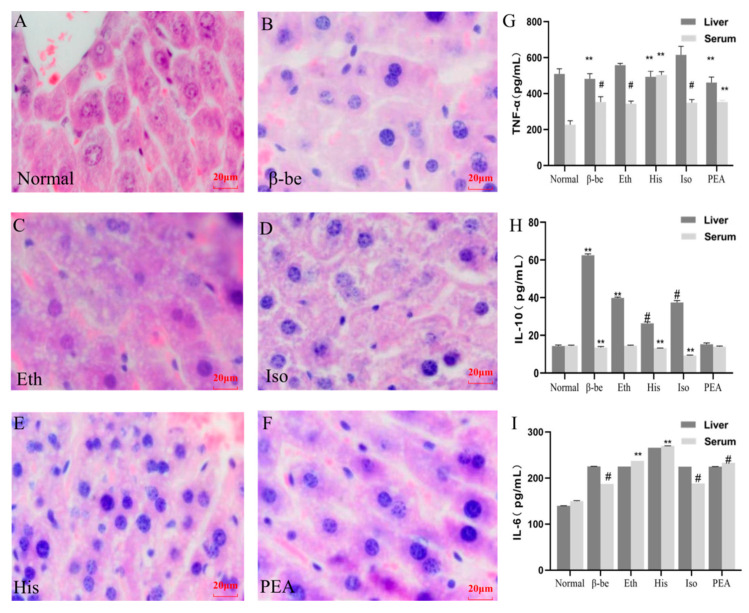
The level of inflammatory cytokines in the liver and serum, and representative histological results using H&E staining. ** *p* < 0.01; ^#^ *p* < 0.05 vs Normal. (**A**) Representative histological results using H&E in Normal. (**B**) Representative histological results using H&E in β-be. (**C**) Representative histological results using H&E in Eth. (**D**) Representative histological results using H&E in His. (**E**) Representative histological results using H&E in Iso. (**F**) Representative histological results using H&E in PEA. (**G**) Liver and Serum TNF-α. (**H**) Liver and Serum IL-10. (**I**) Liver and Serum IL-6.

H&E staining has been performed to assess the effect of different components on the liver. As shown in Figure 2A–F, among the five treated groups, the His group exhibited the most significant difference when compared to the Normal group. Hyperchromatic nuclei and invisible nucleoli were present in the His group. Hepatocytes with two nuclei were common, and other hepatocytes were in the mitotic phase. Liver cells with pathological changes can also be seen in the His group, such as lytic cells, disappeared nucleus, white vacuoles in the cytoplasm, and vacuolated lesions. The lesions observed occurred in more areas in His group compared with other groups (Figure 2A–F). Meanwhile, the His groups showed higher significance in TNF-α and IL-10 levels both in the liver and serum (*p* < 0.01, Figure 2G,H). However, the level of IL-6 had no significant difference among groups (Figure 2I).

### 2.3. Effects of His, PEA, β-Be, and Iso on Pyroptosis in the Liver

**Figure 3:**
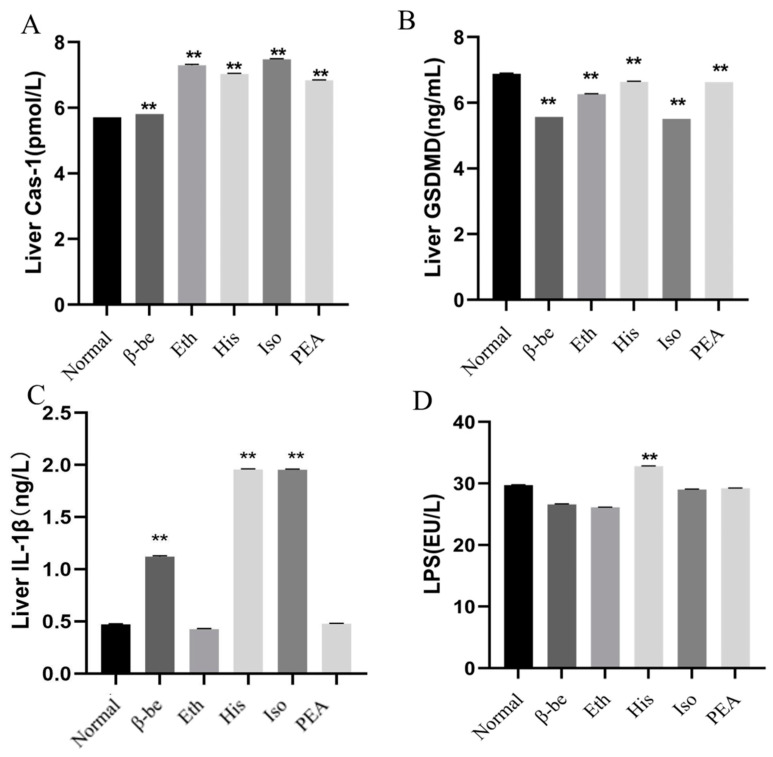
The effects of His, PEA, β-be, and Iso on the pyroptosis pathway and LPS of the gut. ** *p* < 0.01. (**A**) Liver Cas-1. (**B**) Liver GSDMD. (**C**) Liver IL-1β. (**D**) Gut LPS.

As shown in Figure 3A–C, the level of Cas-1, GSDMD, and IL-1β in the His group were significantly enhanced compared with those in the other groups (*p* < 0.01). A similar tendency was seen with the level of Cas-1 and IL-1β in the Iso group. The level of gut LPS was presented in Figure 3D, and we found that the LPS level was significantly higher in the His group than in the other groups (*p* < 0.01).

### 2.4. Effects of His, PEA, β-Be, and Iso on Gut Microbial Community Structure and Composition

**Figure 4:**
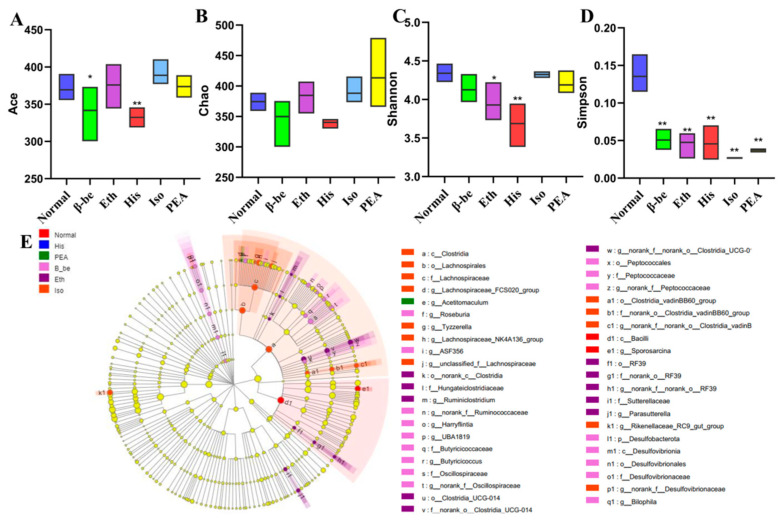
The effects of His, PEA, β-be, and Iso on the Alpha diversity of gut microbiome in mice. * *p* < 0.05; ** *p* < 0.01. (**A**) Ace index. (**B**) Shannon index. (**C**) Chao index. (**D**) Simpson index. (**E**) Biological cladograms.

**Figure 5:**
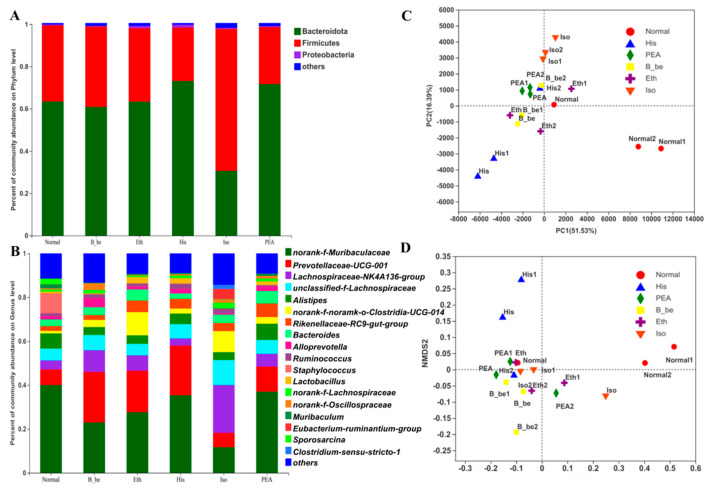
The effects of His, PEA, β-be, and Iso on the community composition amd Beta diversity of gut microbiome in mice. (**A**)The relative abundance plot in bacterial phyla level of the gut microbiota. (**B**) The relative abundance plot in bacterial genera of the gut microbiota. (**C**) Principal coordinate analysis. (**D**) Clustering analysis.

Total DNA was extracted from the feces samples and the 16S rRNA gene of V3–V4 was amplified by PCR. Sequencing of PCR products was performed by using the Illumina MiSeq platform at Majorbio Bio-Pharm Technology Co. Ltd. (Shanghai, China). After removing the low-quality and chimeric sequences, 780,661 high-quality sequencing reads were generated from feces samples. A total of 613 operational taxonomic units (OTUs) were generated from the high-quality sequences at 97% sequence. Ace and Simpson indexes of the gut microbiome in the His group and the β-be group significantly decreased (Figure 4A,D). Shannon index of the gut microbiome in the His group and the Eth group significantly decreased (Figure 4C). However, there is no significance of the Chao index between the treated groups and the Normal group (Figure 4B). LEfse analysis further confirmed the difference of gut microbial structures between all groups (Figure 4E). At the phyla level, there were 19 phyla were identified. The dominant phylum with relative abundance > 1% was Bacteroides (72.75%), Firmicutes (25.09%), and Proteobacteria (1.39%) (Figure 5A) in all groups. At the genus level, there were 213 genera were identified. The dominant genera were considered as norank_f__Muribaculaceae (32.47%), Prevotellaceae_UCG-001 (22.61%), Lachnospiraceae_NK4A136_group (3.24%), unclassified_f__Lachnospiraceae (6.54%), and Alistipes (4.86%) (Figure 5B). We found that the His group decreased the relative abundance of norank_f__Muribaculaceae compared with that of the Normal group. The His, Eth, β-be, PEA group significantly increased the relative abundance of Prevotellaceae_UCG_001 (*p* < 0.01). The gut microbiome composition in His group mice differed substantially from those in the mice in Iso, Eth, and PEA group (Figure 5C,D).

### 2.5. Correlation among Gut Microbiota, Liver Pyroptosis and Microbial Metabolism

**Figure 6:**
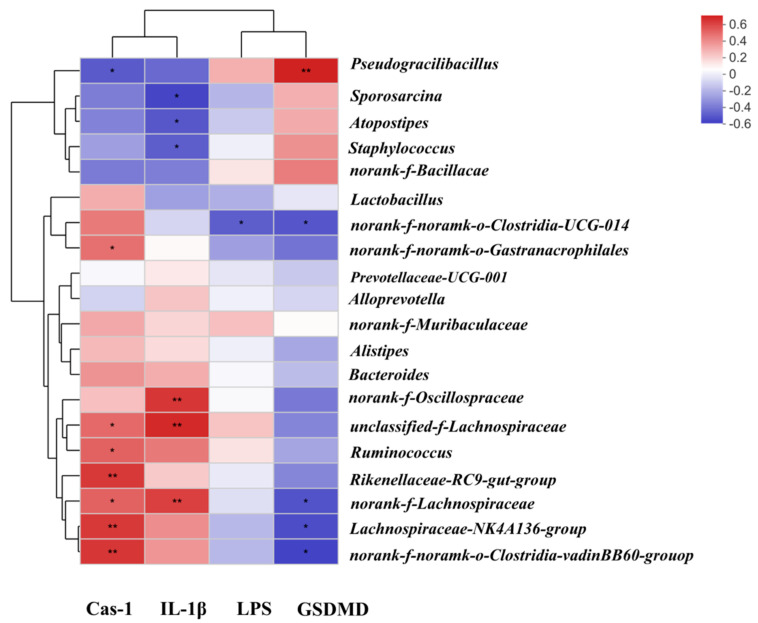
The Spearman’s correlation analysis among gut microbiota, LPS and the levels of Cas-1, GSDMD and IL-1β. * *p* < 0.05; ** *p* < 0.01.

In order to explore the potential relationship between gut and liver, we applied the correlation analysis among gut microbiota, gut LPS, and the levels of IL-1β, Cas-1, and GSDMD through Spearman correlation analysis (Figure 6). As shown in the figure, the relative abundance of Pseudogracbacilus negatively correlated with the level of Cas-1 (*p* < 0.05), but significant positively correlated with the level of GSDMD (*p* < 0.01); the relative abundance of Sporosarcina negatively correlated with the levels of gut LPS and IL-1β (*p* < 0.05); the relative abundance of norank_f_Lachnospiracae negatively correlated with the level of GSDMD but positively correlated with the level of Cas-1 and IL-1β (*p* < 0.05; *p* < 0.01, respectively). We also found that the relative abundance of Lachnospiraceae_NK4A136_group negatively correlated with the level of GSDMD (*p* < 0.05) and positively correlated with the level of Cas-1 (*p* < 0.05).

## 3. Discussion

The mice model is one of the commonly used models to investigate liver damage and has long been used to evaluate the pathological process of alcoholic liver disease (ALD) [17], nonalcoholic liver disease (NALD) [11], and other liver injuries [10]. Compared with gavage, intraperitoneal injection greatly shortens the modeling cycle, but there is a higher risk of death concurrently [18]. In the present study, we investigated the effect of four main components in Huangjiu, His, PEA, β-be, and Iso, on liver pyroptosis by oral treatment in mice. We first investigated the effects of four components on body weight, liver weight, and level of oxidative stress in serum and liver of mice. Simultaneously, the level of inflammatory cytokines in the liver and serum were examined. Results have shown that treatment with four components, especially the His, significantly stimulated the level of oxidative stress and inflammatory cytokines both in serum and liver. In order to explore the underlying mechanism, we performed gut microbiota analysis and pyroptosis pathway assay and found the gut microbial community structure and composition has been changed, resulting in the increase of inflammatory LPS and subsequent pyroptosis of liver cells. Correlation analysis indicated the possible relationship between oral treatment, gut microbiota metabolism, and liver pyroptosis.

Alcohol can induce the increase of endotoxin level, activate Kupffer cells, and trigger the excessive production of TNF-α, IL-1β, and IL-6, which participate in the pathogenesis of liver diseases [19]. TNF-α could induce SREBP1 expression, resulting in hepatic steatosis [20]; IL-1β and IL-6 are involved in hepatic steatosis and inflammation, which causes hepatic disease [21]. In this study, we found that besides ethanol, the expression of TNF-α, IL-1β, and IL-6 in other treated groups increased significantly, which might trigger liver inflammation, directly or indirectly.

Pyroptosis is a programmed cell death characterized by the formation of pores on the plasma membrane, cell swelling, and plasma membrane rupture, similar to necrosis rather than apoptosis [22]. For a long time, IL-1β has been considered an important pyrogen. IL-1β affects the body’s innate and adaptive immunity through different mechanisms, so as to significantly enhance the body’s response to infectious diseases [23]. Although pyroptosis in host defense is largely unclear, it has been proved to inhibit the replication and proliferation of microorganisms in vivo [24]. The proinflammatory effect of IL-1β and pyroptosis could induce autoimmune and inflammatory diseases [25]. As recently reported, Gasdermin D (GSDMD) protein was presented in nigericin-induced NLRP3 inflammasomes, which act on the prediction site of inflammatory activation and participate in pyroptosis and IL-1β secretion [14]. In the present study, the level of GSDMD and IL-1β of the liver in the His group both increased significantly, indicating that pyroptosis caused by His occurred in the liver.

Intestinal homeostasis is maintained by the interrelationship between the gut microbiota, intestinal barrier, and immune system. Imbalance in the gut microbiome often tends to result in the destruction of the intestinal barrier and immune system [26]. Under normal physiological conditions, intestinal epithelial cells, microbiota, and immune cells support the steady-state of the intestinal system collaboratively. Intestinal epithelial cells receive signals from the microbiota, such as microbial metabolites (LPS, short-chain fatty acids, etc.) or the microbes per se, so as to maintain the normal physiological function of the mucosal barrier [27]. LPS is associated with excessive inflammatory response, mainly activating NF*κ*B by binding to peripheral blood macrophages or microglia TLR4/CD14 complex, increasing the production of cytokines such as IL-6 and TNF-α to cause an inflammatory response [28]. In this study, the level of the gut LPS was observed to increase significantly in the His group. Meanwhile, the level of liver IL-6 and TNF-α in the His group increased either, which means the increase of gut LPS might cause liver inflammation, connecting the gut microbiota change and the liver pyroptosis. Nevertheless, the gut microbiota could also regulate host immune function through the action of metabolites or endotoxin [29]. At the same time, immune cells can directly or indirectly affect the growth of the microbiota by releasing cytokines or chemokines [30].

To further explore the underlying mechanism, the effects of His, PEA, β-be, and Iso on the gut microbiota were assessed by 16S rRNA sequencing. We found that His treatment increased the relative abundance of Proteobacteria at the phylum level, which was considered as a microbial marker for dysbiosis [31]. At the genus level, His treatment enriched the relative abundance of *Lachnospiraceae*_*NK4A136*_*group* and *norank*_*f*_*Lachnospiracae*, which belong to the *Lachnospiraceae* family, and can also be enriched by the Fubrick tea in the previous study [32]. Through the correlation analysis among gut microbiota, LPS, and the levels of IL-1β, Cas-1 and GSDMD, we found that the relative abundance of *Lachnospiraceae*_*NK4A136*_*group* negatively correlated with the level of GSDMD (*p* < 0.05) and positively correlated with the level of Cas-1 (*p* < 0.05). *Lachnospiraceae*_*NK4A136*_*group* was reported to negatively correlate with obesity [33]. In our study, the body weight of mice in His group was decreased, and we speculated that *Lachnospiraceae*_*NK4A136*_*group* caused pyroptosis and inflammation in the liver, resulting in weight loss.

**Figure 7:**
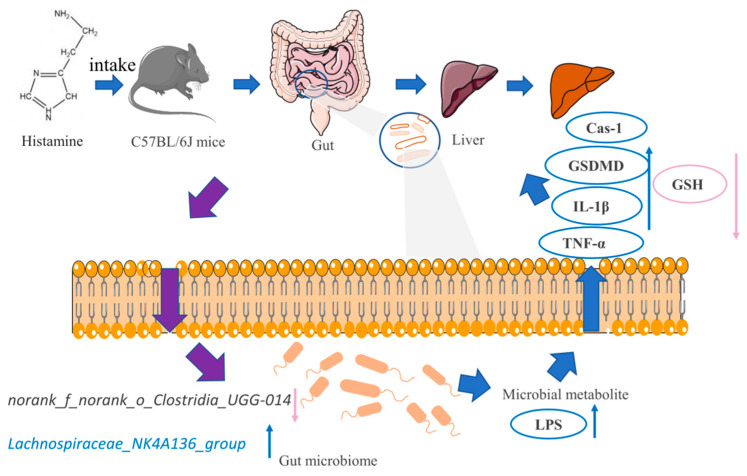
A schematic model shows the mechanism of liver pyroptosis caused by oral histamine. Gut microbiota, oxidative stress, and inflammation are all affected.

In conclusion, the treatment of four main components in Huangjiu, especially the histamine, caused metabolic disorder, oxidative stress, liver pyroptosis, and inflammation via regulating gut microbiota and its metabolism. The possible mechanism was summarized and illuminated in Figure 7. To sum up, oral histamine treatment caused changes in the gut microbiota structure and composition, especially *Lachnospiraceae*_*NK4A136*_*group* and *norank_f_norank_o_Clostridia_UGG-014*, resulting in the increase of inflammatory LPS, which subsequently caused oxidative stress and inflammatory damage to the liver, further lead to liver pyroptosis and may even cause health risks. Although the present study analyzed the destructive effect of main components in Huangjiu on liver and investigated the possible underlying mechanism, there were still some possible limitations in this study. What is the exact source of gut LPS and how the main components in Huangjiu triggered its promotion? Is there any synergetic or antagonistic effect of the four main components? There are too many unresolved questions to be answered. Further studies focusing on the comprehensive effect study of combination of main components, the effective removal of harmful components, as well as other possible harmful effects study of Huangjiu exert on human health, are needed. Overall, the study revealed a possible relationship between gut microbiota and liver metabolism, and the obtained results might help guiding the establishment of a recommended limiting standard of harmful components in Huangjiu, which shed light on the gut-liver axis research and contribute to the development of Huangjiu industry, and should be further investigated.

## 4. Materials and Methods

### 4.1. Materials

His, PEA, β-be, and Iso were purchased from Beijing Pinellia Technology Development Co., Ltd. (Beijing, China). Paraformaldehyde (DPEC) and phosphate-buffered saline (PBS) were obtained from Shanghai Aladdin Bio-Chem Technology Co., LTD (Shanghai, China). The reduced glutathione (GSH), malondialdehyde (MDA), superoxide dismutase (SOD), assay kits were purchased from Nanjing Jiancheng Bioengineering Institute (Nanjing, China). The ELISA kits for interleukin (IL)-1β, interleukin (IL)-6, interleukin (IL)-10, tumor necrosis factor (TNF)-α, Caspase-1(Cas-1), Gasdermin D (GSDMD), and lipopolysaccharides (LPS) were purchased from Enzyme-linked Biotechnology (Shanghai, China), Dakewe Biotech (Beijing, China), R&D Systems, Nanjing Jiancheng Bioengineering Institute (Nanjing China), and Jiangsu Meimian industrial Co., Ltd. (Yancheng, China). All other reagents used in this essay were of analytical grade.

### 4.2. Animals and Experimental Treatment

Thirty-six male C57BL/6J mice (6–8 weeks old, 25–30 g, Specific Pathogen Free) and fodder were obtained from Beijing Vital River Laboratory Animal Center [SCXK(Jing)2016-0006]. The mice were acclimated for 1 week before the experiments. The mice were housed in a room with a controlled temperature (22 ± 2 °C), humidity (65% ± 5%), and 12 h dark/light cycle. After the acclimation period, mice were randomly divided into six groups(n = 6): Normal (normal control: feed with normal saline,15 mL/kg BW), Eth (15% (*v*/*v*) ethanol solution, 15 mL/kg BW), His (histamine dissolved in 15% (*v*/*v*) ethanol solution, 15 mL/kg BW), PEA (phenethylamine prepared in 15% (*v*/*v*) ethanol solution, 15 mL/kg BW), β-be (β-benzyl ethanol dissolved in 15% (*v*/*v*) ethanol solution, 15 mL/kg BW), Iso (isopentanol prepared in 15% (*v*/*v*) ethanol solution, 15 mL/kg BW) (Figure S1). The dosages of these substances above were set according to Peng et al. [34]. According to the weight of 60 kg adult drink 2 bottles of 500 mL Huangjiu, according to the concentration of each substance in Huangjiu, we calculated the content of substance intake per kg body weight of an adult, and the corresponding dose is determined by the intake per kg body weight of mice. The specific parameters were shown in Table S1. All groups received normal foods and water. The treatment was given by gavage once a day for 2 weeks; body weight was measured every other day.

After the experiment, the mice feces were collected in the sterile sampling tube and frozen at −80 °C for later use. At the end of the experiment, the mice fasted for 12 h were anesthetized and euthanized, and their serum was collected in endotoxin-free tubules. After labeling, the serum samples were centrifuged at 4 °C at 3000× *g* for 10 min and stored at −80 °C for further analysis. After serum collection, the liver and gut tissues of mice were removed and placed in pre-cooled normal saline for rinsing to remove the serum stuck to it separately, and the surface moisture was sucked out with filter paper. It was weighed and cut into pieces, one part was soaked in a preconfigured solution of 4% (*v*/*v*) DPEC (prepared in PBS), the other part was added 9 times 0.9% pre-cooled normal saline to prepare a 10% (*w*/*v*) homogenate, placed in a glass homogenizer for rapid grinding with an ice-cold bath, centrifuged at 12,000× *g* at 4 °C for 15 min, then the supernatant was taken and stored at −80 °C for later use.

### 4.3. Biomarker Analysis of Serum and Liver

Serum and liver malondialdehyde (MDA), reduced glutathione (GSH), superoxide dismutase (SOD) levels were determined using assay kits purchased from Nanjing Jiancheng Bioengineering Institute (Nanjing, China); interleukin (IL)-6, interleukin (IL)-10, tumor necrosis factor (TNF)-α were measured via ELISA kits purchased from Enzyme-linked Biotechnology (Shanghai, China), Dakewe Biotech (Beijing, China), R&D Systems, Nanjing Jiancheng Bioengineering Institute (Nanjing China). All experimental operations have been quantified in accordance with the manufacturer’s instructions.

### 4.4. Histopathological Analysis of Liver Tissues

Liver tissue fixed in 4% DPEC was further dehydrated, embedded in paraffin, sliced in 4 µm thickness, and stained with hematoxylin and eosin (H&E). Subsequent examination of the prepared slides was under a panoramic microscope (3D HITECH Panimal 250, Hungary) [35].

### 4.5. Validation of the Cellular Pyroptosis Pathway

NLRP-3/Cas-1/GSDMD/IL-1β was one of the most important inflammasome-mediated pyroptosis pathways identified recently [14]. Liver Cas-1, GSDMD, and IL-1β levels were measured via ELISA assay kits obtained from Jiangsu Meimian industrial Co., Ltd. (Yancheng, China). All experimental procedures were quantified under the supplier’s instructions.

### 4.6. Determination of LPS in Gut Tissues

Gut LPS level was determined using ELISA assay kits obtained from Jiangsu Meimian industrial Co., Ltd. (Yancheng, China). The experimental procedures were quantified according to the supplier’s instructions.

### 4.7. Analysis of Gut Microbiome

16S rRNA analysis was performed on frozen feces to analyze the gut microbial community structure and composition. Fecal total DNA was extracted and sequenced.

The universal primer pairs 338F and 806R were used to amplify the V3-V4 hypervariable region of the 16S rRNA gene [19]. Purified amplicons were pooled in equimolar and paired-end sequenced on an Illumina MiSeq PE300 platform (Illumina, San Diego, CA, USA) according to the standard protocols by Majorbio Bio-Pharm Technology Co. Ltd. (Shanghai, China). Operational taxonomic units (OTUs) with 97% similarity cutoff were clustered using UPARSE version 7.1 [36], and chimeric sequences were identified and removed. The taxonomy of each OTU representative sequence was analyzed by RDP Classifier version 2.2 [37] against the 16S rRNA database using a confidence threshold of 0.7.

Assessment of alpha diversity by Chao richness, ACE index, Shannon and Simpson diversity indices using Sob’s estimator [38]. Beta diversity at the OTU level was analyzed by principal component analysis (PCA) and non-metric multidimensional scaling anal-ysis (NMDS). The linear discriminant analysis effect size (LEfSe) method was used to identify differentially rich taxa in each treatment group, with the LDA score set at 4.0 [39].

### 4.8. Correlation Analysis

In order to further explore the relationship among His, PEA, β-be, and Iso, gut microbiota, microbial metabolites, and biomarkers mentioned above, Spearman correlation analysis was used to study the relationship between different gut microbiota and LPS, and LPS and biomarkers. If the absolute correlation coefficient is greater than 0.6 and *p* < 0.05, the correlation was considered significant. Heat maps were obtained using R software to visualize the results.

### 4.9. Statistical Analysis

The results are expressed as means ± SD (standard errors of the means) and analyzed using GraphPad Prism 8.0 program (GraphPad Software, San Diego, CA, USA). The data were evaluated using a one-way ANOVA analysis of variance by Ducan’s test, when *p* < 0.05 was considered significant.

## Data Availability

Data are available upon request to corresponding authors. The data presented in this study are available in the article and supplementary data.

## Supplementary Materials

The following supporting information can be downloaded at: https://www.mdpi.com/article/10.3390/ijms23073710/s1.

## References

[1] Li W., Fan G., Fu Z., Wang W., Xu Y., Teng C., Zhang C., Yang R., Sun B., Li X. (2020). Effects of fortification of Daqu with various yeasts on microbial community structure and flavor metabolism. Food. Res. Int..

[2] Zou W., Ye G., Zhang K. (2018). Diversity, function, and application of *Clostridium* in Chinese strong flavor Baijiu ecosystem: A review. J. Food. Sci..

[3] Wu Z., Xu E., Long J., Wang F., Xu X., Jin Z., Jiao A. (2015). Measurement of fermentation parameters of Chinese rice wine using Raman spectroscopy combined with linear and non-linear regression methods. Food. Cont..

[4] Chen S., Xu Y., Qian M.C. (2013). Aroma characterization of Chinese rice wine by gas chromatography-olfactometry, chemical quantitative analysis, and aroma reconstitution. J. Agric. Food. Chem..

[5] Liu S.P., Yu J.X., Wei X.L., Ji Z.W., Zhou Z.L., Meng X.Y., Mao J. (2016). Sequencing-based screening of functional microorganisms to decrease the formation of biogenic amines in Chinese rice wine. Food. Cont..

[6] Sun H., Liu S., Mao J., Yu Z., Lin Z., Mao J. (2020). New insights into the impacts of Huangjiu components on intoxication. Food. Chem..

[7] Krymchantowski A.V., Jevoux C.D.C. (2014). Wine and headache. Headache.

[8] Han H., Jiang Y., Wang M., Melaku M., Liu L., Zhao Y., Everaert N., Yi B., Zhang H. (2021). Intestinal dysbiosis in nonalcoholic fatty liver disease (NAFLD): Focusing on the gut-liver axis. Crit. Rev. Food. Sci. Nutr..

[9] Brandl K., Hartmann P., Jih L.J., Pizzo D.P., Argemi J., Ventura-Cots M., Coulter S., Liddle C., Ling L., Rossi S.J. (2018). Dysregulation of serum bile acids and FGF19 in alcoholic hepatitis. J. Hepatol..

[10] Do M.H., Lee H.H.L., Kim Y., Lee H.-B., Lee E., Park J.H., Park H.-Y. (2021). *Corchorus olitorius* L. ameliorates alcoholic liver disease by regulating gut-liver axis. J. Funct. Foods..

[11] Chu H., Duan Y., Yang L., Schnabl B. (2019). Small metabolites, possible big changes: A microbiota-centered view of non-alcoholic fatty liver disease. Gut.

[12] Sharpton S., Schnabl B., Knight R., Loomba R. (2021). Current concepts, opportunities, and challenges of gut microbiome-based personalized medicine in nonalcoholic fatty liver disease. Cell. Metab..

[13] Friedman E.S., Li Y., Shen T.C.D., Jiang J., Chau L., Adorini L., Babakhani F., Edwards J., Shapiro D., Zhao C. (2018). FXR-dependent modulation of the human small intestinal microbiome by the bile acid derivative obeticholic acid. Gastroenterology.

[14] He W.-T., Wan H., Hu L., Chen P., Wang X., Huang Z., Yang Z.-H., Zhong C.-Q., Han J. (2015). Gasdermin D is an executor of pyroptosis and required for interleukin-1β secretion. Cell. Res..

[15] Ruan J., Wang S., Wang J. (2020). Mechanism and regulation of pyroptosis-mediated in cancer cell death. Chem. Biol. Int..

[16] Zeng C., Wang R., Tan H. (2019). Role of pyroptosis in cardiovascular diseases and its therapeutic implications. Int. J. Biol. Sci..

[17] Seo B., Jeon K., Moon S., Lee K., Kim W.-K., Jeong H., Cha K.H., Lim M.Y., Kang W., Kweon M.-N. (2020). *Roseburia* spp. abundance associates with alcohol consumption in humans and its administration ameliorates alcoholic fatty liver in mice. Cell. Host. Microbe..

[18] Gong J., Szego É.M., Leonov A., Benito E., Becker S., Fischer A., Zweckstetter M., Outeiro T., Schneider A. (2019). Translocator protein-ligand protects against neurodegeneration in the MPTP mouse model of parkinsonism. J. Neurosci..

[19] Yang K., Zhan L., Lu T., Zhou C., Chen X., Dong Y., Lv G., Chen S. (2020). *Dendrobium officinale* polysaccharides protected against ethanol-induced acute liver injury in vivo and *in vitro* via the TLR4/NF-*kappa*B signaling pathway. Cytokine.

[20] Endo M., Masaki T., Seike M., Yoshimatsu H. (2007). TNF-alpha induces hepatic steatosis in mice by enhancing gene expression of sterol regulatory element-binding protein-1c (SREBP-1c). Exp. Bio. Med..

[21] Karatayli E., Hall R.A., Weber S.N., Dooley S., Lammert F. (2019). Effect of alcohol on the interleukin 6-mediated inflammatory response in a new mouse model of acute-on-chronic liver injury. Biochim. Biophys. Acta. Mol. Basis. Dis..

[22] Fink S.L., Cookson B.T. (2005). Apoptosis, pyroptosis, and necrosis: Mechanistic description of dead and dying eukaryotic cells. Infect. Immun..

[23] Netea M.G., Simon A., van de Veerdonk F., Kullberg B.-J., Van der Meer J.W.M., Joosten L.A.B. (2010). IL-1beta processing in host defense: Beyond the inflammasomes. PLoS. Pathog..

[24] Miao E.A., Leaf I.A., Treuting P.M., Mao D.P., Dors M., Sarkar A., Warren S.E., Wewers M.D., Aderem A. (2010). Caspase-1-induced pyroptosis is an innate immune effector mechanism against intracellular bacteria. Nat. Immunol..

[25] Lamkanfi M., Dixit V.M. (2014). Mechanisms and functions of inflammasomes. Cell.

[26] Kayama H., Okumura R., Takeda K. (2020). Interaction between the microbiota, epithelia, and immune cells in the intestine. Annu. Rev. Immunol..

[27] von Moltke J., Ji M., Liang H.-E., Locksley R.M. (2016). Tuft-cell-derived IL-25 regulates an intestinal ILC2-epithelial response circuit. Nature.

[28] Zhan X., Stamova B., Sharp F.R. (2018). Lipopolysaccharide associates with amyloid plaques, neurons and oligodendrocytes in Alzheimer’s disease brain: A review. Front. Aging. Neurosci..

[29] Round J.L., Mazmanian S.K. (2009). The gut microbiota shapes intestinal immune responses during health and disease. Nat. Rev. Immunol..

[30] Levy M., Kolodziejczyk A.A., Thaiss C.A., Elinav E. (2017). Dysbiosis and the immune system. Nat. Rev. Immunol..

[31] Shin N.-R., Whon T.W., Bae J.-W. (2015). *Proteobacteria*: Microbial signature of dysbiosis in gut microbiota. Trends. Biotechnol..

[32] Jing N., Liu X., Jin M., Yang X., Hu X., Li C., Zhao K. (2020). Fubrick tea attenuates high-fat diet induced fat deposition and metabolic disorder by regulating gut microbiota and caffeine metabolism. Food. Funct..

[33] Tong A., Hu R., Wu L., Lv X., Li X., Zhao L., Liu B. (2020). Ganoderma polysaccharide and chitosan synergistically ameliorate lipid metabolic disorders and modulate gut microbiota composition in high fat diet-fed golden hamsters. J. Food. Biochem..

[34] Peng L., Liu S., Ji Z., Chen S., Mao J. (2019). Structure characterization of polysaccharide isolated from Huangjiu and its anti-inflammatory activity through MAPK signaling. Int. J. Food. Sci. Tech..

[35] Mehmood A., Zhao L., Wang C., Hossen I., Raka R.N., Zhang H. (2019). Stevia residue extract increases intestinal uric acid excretion via interactions with intestinal urate transporters in hyperuricemic mice. Food. Funct..

[36] Wang Q., Garrity G.M., Tiedje J.M., Cole J.R. (2007). Naive Bayesian classifier for rapid assignment of rRNA sequences into the new bacterial taxonomy. Appl. Environ. Microbiol..

[37] Edgar R.C. (2013). UPARSE: Highly accurate OTU sequences from microbial amplicon reads. Nat. Methods.

[38] Couvigny B., de Wouters T., Kaci G., Jacouton E., Delorme C., Dore J., Renault P., Blottière H.M., Guédon E., Lapaque N. (2015). Commensal streptococcus salivarius modulates PPAR gamma transcriptional activity in human intestinal epithelial cells. PLoS ONE.

[39] Schloss P.D., Westcott S.L., Ryabin T., Hall J.R., Hartmann M., Hollister E.B., Lesniewski R.A., Oakley B.B., Parks D.H., Robinson C.J. (2009). Introducing mothur: Open-source, platform-independent, community-supported software for describing and comparing microbial communities. Appl. Environ. Microbiol..

