# Supplementary material for: Histamine Causes Pyroptosis of Liver by Regulating Gut-Liver Axis in Mice
Source: Int J Mol Sci. 2022 Mar 28;23(7):3710. doi: 10.3390/ijms23073710 (PMC8998596; doi:10.3390/ijms23073710)
Supplement: Supplementary file 1 [file ijms-23-03710-s001.zip › ijms-1651781-supplementary.pdf]

Supplementary Material

Histamine causes pyroptosis of liver by regulating gut-liver axis in mice

Qiaoqiao Luo[1], Ruoyu Shi[1], Yutong Liu[1], Libo Huang[2], Wei Chen[1*] and Chengtao Wang[1*]

[1] Beijing Advanced Innovation Center for Food Nutrition and Human Health, Beijing Engineering and Technology Research Center of Food Additives, School of Food and Health, Beijing Technology and Business University, Beijing 100048, China; Luoqiaoqiao7469@163.com (Q.L.); shiruoy@126.com (R.S.); liuyt128@126.com (Y.L.).

[2] College of Animal Science and Technology, Shandong Agricultural University, Taian 271000, China; huanglibo@sdau.edu.cn (L.H)

* Correspondence:

weichen@btbu.edu.cn (W. Chen);

wangchengtao@th.btbu.edu.cn (C. Wang).

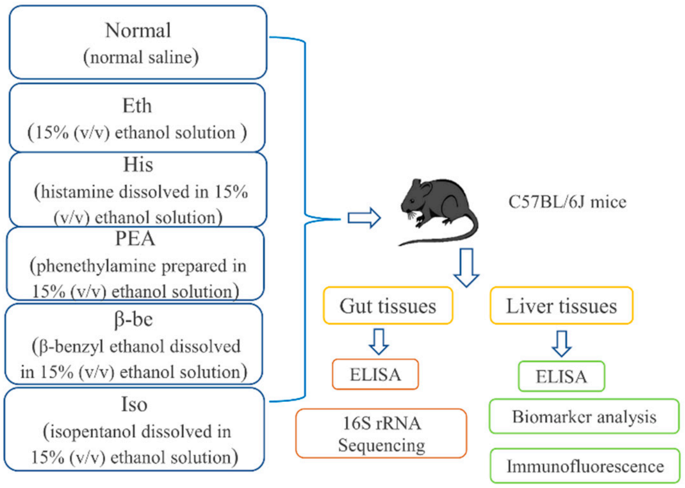

Figure S1 A brief summary of the experimental treatment

Table S1 The dosage of each substance in mice

compounds	the content in Huangjiu（mg/L）	the dosage of mice（mg/25g.bw）
His	17.00	0.14
PEA	117.97	0.98
Iso	189.26	1.58
β-be	128.61	1.07
